# Evaluation of Subclinical Left Ventricular Systolic Dysfunction in Chronic Asymptomatic Alcoholics by Speckle Tracking Echocardiography

**DOI:** 10.1155/2017/6582568

**Published:** 2017-03-29

**Authors:** Murathan Kucuk, Can Ramazan Oncel, Aytul Belgi Yıldırım, Fatih Canan, Mehmet Murat Kuloglu

**Affiliations:** ^1^Akdeniz University Medical Faculty, Department of Cardiology, Antalya, Turkey; ^2^Ataturk State Hospital, Department of Cardiology, Antalya, Turkey; ^3^Akdeniz University Medical Faculty, Department of Psychiatry, Antalya, Turkey

## Abstract

By using two-dimensional speckle tracking echocardiography, we aimed to investigate the structural and functional changes on myocardium in chronic asymptomatic alcoholics without any cardiovascular disease. Forty-one consecutive asymptomatic male alcoholics who were admitted to the outpatient alcoholism unit and 30 age matched healthy male volunteers selected as the control group were enrolled in the study. The study group were investigated by using standard two-dimensional echocardiography and speckle tracking echocardiography. The left ventricular (LV) global longitudinal strain and LV global circumferential strain were significantly lower in alcoholics when compared with control subjects. There was no difference in global radial strain between the two groups. To demonstrate the effect of total life time dose of ethanol (TLDE) on echocardiographic abnormalities, we assessed the correlation analysis. There was a nonsignificant weak correlation between global LV circumferential strain and TLDE (*r* = 0.27, *p* = 0.083). Speckle tracking echocardiography derived left ventricular systolic function was impaired in chronic alcoholic patients when compared with healthy controls.

## 1. Introduction

Chronic excessive alcohol consumption is related with cardiomyopathy where alcoholic cardiomyopathy has been seen in about 10% of alcoholics and prevalence ranges from 23% to 40% [1].

Transthoracic echocardiography can detect mild left ventricular hypertrophy, diastolic dysfunction, and left ventricular dilatation in asymptomatic alcoholics [2]. Although reduced left ventricular ejection fraction can be documented by conventional echocardiography, it is important to find a method for the detection of subclinical LV systolic dysfunction in these patients. Predicting early cardiac toxicity of chronic alcohol consumption in patients at high risk for developing alcoholic cardiomyopathy will be helpful, since an early withdrawal of alcohol may improve left ventricular systolic function and prognosis in this group.

Our study aims to analyze the structural and functional changes on myocardium using two-dimensional speckle tracking echocardiography in chronic asymptomatic alcoholics without any cardiovascular disease and any disease which could impair myocardial functions.

## 2. Methods

Forty-one consecutive asymptomatic male alcoholics who were admitted to the outpatient alcoholism unit which is located in Mediterranean region of Turkey were enrolled in this study. Thirty age matched healthy male volunteers were selected as the control group. None of them had any history, signs, or symptoms of cardiovascular diseases.

The exclusion criteria were a history of congenital heart disease, hypertension, diabetes mellitus, coronary artery disease, valvular heart disease, atrial fibrillation, and systemic and metabolic disease such as chronic liver disease or renal dysfunction. All the patients were questioned about the amount and duration of alcohol consumption.

Total lifetime dose of ethanol (TLDE) was calculated by first multiplying the daily consumption of ethanol by the number of days of the periods of exposure to alcohol and then dividing the product by the body weight of the patient [3].

### 2.1. Echocardiography

Echocardiographic examinations including conventional, pulsed Doppler, tissue Doppler, and speckle tracking echocardiography were performed in 41 alcoholics and 30 age matched controls. Echocardiographic study was done by ultrasound machine GE-Vingmed vivid 7 system (GE-Vingmed Ultrasound AS, Horten, Norway) and 3S-RS (3.5 mhz) probe.

Speckle tracking echocardiography analysis was performed from apical and parasternal short axis views, respectively. Standard grayscale 2D images were obtained at a frame rate of 70–90 frames/s during three cardiac cycles and software package (Echopac PC, version 6.0, GE healthcare) was used for offline analysis. Endocardial border of the left ventricle was traced manually from the end systolic frame. The software automatically detected the epicardial border and created a region of interest which was adjusted manually to include the entire myocardial wall. Apical and short axis views of left ventricle were processed by software to select natural acoustic markers. Then these markers were tracked frame by frame during cardiac cycle to measure strain and strain rate of left ventricle at any point of the myocardium. To obtain optimal tracking, necessary corrections were done for the quality tracking verification and region of interest modification. All echocardiographic measurements were done according to the guidelines of American Society of Echocardiography.

### 2.2. Clinical and Laboratory Evaluations

After a complete physical examination, anthropometric measurements of height and weight were done and body mass index (BMI, kg/m^2^) and body surface area (BSA, m^2^) of all subjects were calculated. Venous blood count was obtained from all subjects on admission as a routine part of clinical examination. A detailed questionnaire was applied to evaluate the real dose of alcohol consumed and then confirmed by family members because of the known characteristics of alcoholics.

### 2.3. Statistical Analysis

Continuous and categorical variables were expressed as mean ± standard deviation (SD) and percentages, respectively. The Kolmogorov-Smirnov test was used to verify the normality of the distribution of continuous variables. Continuous variables were compared using one-way ANOVA test. Categorical variables were compared using chi-square test (*χ*2) or Fisher's exact test, as appropriate. Spearman's test was used for correlation analysis. A *p* value of less than 0.05 was regarded significant for all analyses. Analyses were performed with PASW 18 (SPSS/IBM, Chicago, IL, USA) software and two-tailed *p* value less than 0.05 was considered statistically significant.

### 2.4. Inter- and Intraobserver Variability

We reported strain measurements in 20 random alcoholics or control subjects three weeks after the first evaluation for determining intraobserver variability. Also these measurements were done by another observer for determining interobserver variability. The Bland-Altman analysis was calculated for interobserver and intraobserver variability.

## 3. Results

Thirty healthy subjects and 41 alcoholics were evaluated in the study. Detailed demographic, clinical, and biochemical variables of two groups are shown in Table 1. The M mode, two-dimensional, pulse Doppler, and tissue Doppler echocardiographic parameters of two groups were given in Table 2.

Left ventricular ejection fraction did not show any difference between healthy subjects and the alcoholics. Compared to controls, alcoholic group had a significant difference in left atrium diameter (31.83 ± 0.47, 34.13 ± 0.56, *p* = 0.003). There were no differences in LV mass index, EDD, ESD, E/A ratio, IVRT, and IVCT among two groups. Deceleration time was longer in alcoholics (186.14 ± 4.49, 204 ± 6.17, *p* = 0.019).

Alcoholic patients had consumed a mean daily dose of ethanol of 186.64 ± 7 g over a period of 20.38 ± 1.37 years with a mean TLDE of 19.94 ± 12.17 kg ethanol/kg body weight.

### 3.1. Left Ventricular Deformation Analysis

The LV global longitudinal strain and LV global circumferential strain were significantly lower in alcoholics when compared with control subjects. There was no difference in global radial strain between two groups. Strain measurements of two groups were given in Table 3. To demonstrate the effect of TLDE on echocardiographic abnormalities, we used the correlation analysis. There was a nonsignificant weak correlation between global LV circumferential strain and TLDE (*r* = 0.27, *p* = 0.083).

Using two-dimensional speckle tracking echocardiography to evaluate alcoholic cardiac damage, inter- and intraobserver variability results showed a good reproducibility and small variability.

## 4. Discussion

In our study we demonstrated that left ventricular global longitudinal strain and left ventricular global circumferential strain were deteriorated in chronic alcoholic patients. It has been shown that global longitudinal strain is a quantitative index for global left ventricle function [4]. Two-dimensional speckle tracking echocardiography allowed us to detect early systolic dysfunction in chronic alcoholic patients with a preserved LVEF.

Chronic alcoholic consumption causes several histological and cellular changes in the myocardium. These changes include myocyte death, intracellular dysfunction, deterioration of mitochondrial ultrastructure, and sarcoplasmic reticulum [5]. Also it has been shown that cardiac myofibril shortening, composition of contractile proteins, and calcium homeostasis were adversely affected by excessive long term alcohol consumption [6]. These myocardial changes could lead to alcoholic cardiomyopathy later. In previous studies alcoholic patients with alteration in myocardial structure and function had a history of consuming >90 g/d of alcohol for 15 years [7–11]. It has been suggested that duration of daily alcohol consumption is more important than amount of daily alcohol consumption in developing heart failure [12, 13]. In our present study, alcoholic patients have consumed a mean daily dose of ethanol 186.64 ± 7 g over a period of 20.38 ± 1.37 years.

The subclinical cardiac damage in chronic alcoholic patients was investigated in various studies using conventional and tissue Doppler echocardiography. Previous clinical studies evaluating the effect of chronic alcohol consumption on LV systolic and diastolic functions showed conflicting results. Impaired ejection fraction, preserved ejection fraction, and normal and impaired LV filling parameters were reported as a result in these studies [14–19].

Although the natural course of chronic alcoholic cardiomyopathy could not be defined clearly, it has been shown that abstaining from alcohol is associated with improvement in ejection fraction [20, 21]. As most of the alcoholics remain asymptomatic in the early stage of disease progression, early detection of subclinical LV dysfunction may lead to identifying patients at higher risk for heart failure and lead to interventions which would improve the health of alcoholic patients in entire society.

Two-dimensional speckle tracking echocardiography (STE) is a new noninvasive ultrasound imaging technique which allows measuring LV rotation, torsion, and strain as an evaluation of global and regional myocardial function. Speckle tracking echocardiography is angle independent and is less affected by artifacts, acoustic noise, and translational movements [22, 23]. Speckle tracking echocardiographic parameters have been shown to detect subclinical regional and global myocardial dysfunction at an early stage in contrast to evaluation of ejection fraction by conventional methods [24, 25].

In our study all asymptomatic alcoholics had normal cardiac structure and ejection fraction, except that alcoholic patients had a significant difference in left atrial diameter. Similar to our study, Singh et al. demonstrated that modest levels of alcohol consumption were associated with significant left atrium enlargement [26]. Different effects of alcohol consumption on lipoproteins in human and animal studies have been reported before. In previous studies, it has been shown that alcohol consumption is associated with increased levels of both HDL-2 and HDL-3 [27, 28]. Although we could not measure HDL subfractions in our current study, we found that HDL cholesterol levels were significantly higher in alcoholics than healthy controls. Experimental studies showed conflicting results about effects of alcohol on the inflammatory process. In epidemiologic studies it has been demonstrated that moderate alcohol consumption is related with low C-reactive protein (CRP) levels whereas high alcohol intake is associated with increased CRP levels. In the present study, increased CRP levels were observed in alcoholic patients group similar to previous studies [29, 30].

Our present study showed the limitations of conventional echocardiographic evaluation of early deterioration in cardiac systolic function and the value of speckle tracking echocardiography in subclinic alcoholic cardiomyopathy. The novel echocardiographic modalities would allow for demonstration of heavy alcohol consumption outcomes on systolic function.

The gross limitation of our study was the small sample size. Only male subjects were included in our study; therefore the effects of chronic alcoholism on LV functions in women could not be evaluated. Since there are no clear data related to classification, we could not group patients according to amount and duration of alcohol consumption for subgroup analysis. Clinical follow-up was not planned so the effect of subclinical dysfunction on cardiovascular prognosis remains unclear. The importance of these subclinical findings should be determined with a well-designed prospective study including cardiovascular prognosis.

## 5. Conclusions

Even with the abovementioned limitations we can conclude that speckle tracking echocardiography derived left ventricular systolic function is impaired in chronic alcoholic patients when compared with healthy controls.

## Figures and Tables

**Table 1:** Demographic, clinical, and biochemical variables of alcoholic patients and controls.

	Alcoholic patients *N* = 41	Healthy controls *N* = 30	*p*
Age (years)	43.17 ± 1.4	38.0 ± 1.73	0.022
BMI (kg/m^2^)	24.79 ± 0.68	26.60 ± 0.82	0.093
Body surface area (m^2^)	1.89 ± 0.03	1.96 ± 0.03	0.123
Systolic blood pressure (mmHg)	119.29 ± 1.7	118.77 ± 1.54	0.829
Diastolic blood pressure (mmHg)	77.86 ± 1.0	73.83 ± 1.21	0.012
AST (U/L)	65.33 ± 12.47	22.39 ± 1.21	0.005
ALT (U/L)	62.54 ± 10.96	26.73 ± 2.06	0.008
TChol (mg/dL)	207.92 ± 6.92	182.70 ± 8.39	0.023
HDL-Chol (mg/dL)	59.89 ± 3.20	39.66 ± 1.76	0.000
LDL-Chol (mg/dL)	118.32 ± 5.67	110.70 ± 6.45	0.381
TG (mg/dL)	185.61 ± 17.9	167.17 ± 12.54	0.439
Glucose (mg/dL)	86.76 ± 1.47	87.80 ± 1.64	0.641
ESR (mm/hour)	13.31 ± 0.93	5.80 ± 1.06	0.000
CRP (mg/dL)	0.89 ± 0.08	0.18 ± 0.04	0.000
Hemoglobin, g/L	14.78 ± 0.22	15.33 ± 0.15	0.067
WBC count	8585.71 ± 449	7668 ± 342	0.135
RDW	13.91 ± 0.19	13.67 ± 0.16	0.369
MPV	8.58 ± 0.19	7.71 ± 0.23	0.004
Neutrophil count	5305.52 ± 355	4386.33 ± 242	0.054
Lymphocyte count	2221.95 ± 115	2386.33 ± 136	0.361
Creatinine, mg/dl	0.79 ± 0.01	0.86 ± 0.02	0.008
Duration of heavy drinking year (years)	20.38 ± 1.37	0	—
Daily ethanol consumption (g)	186.64 ± 7.0	0	—
TLDE (kg ethanol/kg body weight)	19.94 ± 12.17	0	—

BMI: body mass index, CRP: C-reactive protein, ESR: erythrocyte sedimentation rate, WBC: white blood cell, RDW: red cell distribution width, MPV: mean platelet volume, AST: aspartic acid transaminase, ALT: alanine transaminase, TLDE: total lifetime dose of ethanol.

Data are expressed as mean ± standard deviation.

**Table 2:** Conventional echocardiographic data of alcoholic patients and controls.

	Control group *n* = 30	Alcoholic group *n* = 41	*p*
ARD (mm)	29.67 ± 1.40	30.33 ± 1.40	0.252
LV-EDD (mm)	46.80 ± 1.77	48.21 ± 1.61	0.150
LV-ESD (mm)	28.33 ± 1.71	29.17 ± 1.57	0.359
EF%	70.33 ± 1.06	69.74 ± 1.03	0.695
FS%	39.93 ± 2.87	39.71 ± 2.86	0.862
IVS (cm)	0.95 ± 0.16	0.96 ± 0.15	0.457
PWT (cm)	0.94 ± 0.20	0.90 ± 0.20	0.219
LAD (mm)	31.83 ± 0.47	34.13 ± 0.56	0.003
Mit E wave velocity (m/s)	0.77 ± 0.16	0.78 ± 0.16	0.775
Mit E DT (ms)	204.01 ± 6.17	186.14 ± 4.49	0.019
Mit A wave velocity (m/s)	0.71 ± 0.16	0.64 ± 0.12	0.03
E/A	1.14 ± 0.37	1.28 ± 0.39	0.120
ET (msn)	250.20 ± 28.84	253.76 ± 24.01	0.439
IVRT (msn)	78.97 ± 21.04	76.98 ± 20.58	0.393
IVCT (msn)	57.17 ± 15.15	56.62 ± 22.39	0.772
MPI	0.55 ± 0.15	0.53 ± 0.18	0.210

ARD: aortic root diameter, LV: left ventricular, EDD: end diastolic diameter, ESD: end systolic diameter, EF: ejection fraction, FS: fractional shortening, IVS: septal thickness at end diastole, PWT: posterior wall thickness at end diastole, LAD: left atrium diameter, DT: deceleration time, ET: ejection time, IVRT: isovolumetric relaxation time, IVCT: isovolumetric contraction time, MPI: Myocardial Performance Index.

**Table 3:** Left ventricular strain measurements of alcoholic patients and controls.

	Control group *n* = 30	Alcoholic group *n* = 41	*p*
LV global longitudinal strain (%)	−19.97 ± −7.37	−17.97 ± −6.38	0.001
LV global circumferential strain (%)	−21.18 ± −3.78	−17.17 ± −4.64	0.000
LV global radial strain (%)	46.76 ± 6.94	45.03 ± 7.63	0.596
